# Use of Cell-SELEX to Generate DNA Aptamers as Molecular Probes of HPV-Associated Cervical Cancer Cells

**DOI:** 10.1371/journal.pone.0036103

**Published:** 2012-04-20

**Authors:** Jessica C. Graham, Helmut Zarbl

**Affiliations:** 1 Department of Environmental and Occupational Medicine, Robert Wood Johnson, Medical School, University of Medicine and Dentistry of New Jersey, Piscataway, New Jersey, United States of America; 2 Program in Carcinogenesis and Chemoprevention, Division of Public Health Sciences, Cancer Institute of New Jersey, New Brunswick, New Jersey, United States of America; 3 NIEHS Center for Environmental Exposures and Disease, Environmental and Occupational Health Sciences Institute, University of Medicine and Dentistry of New Jersey, Piscataway, New Jersey, United States of America; Institut Gustave Roussy, France

## Abstract

**Background:**

Disease-specific biomarkers are an important tool for the timely and effective management of pathological conditions, including determination of susceptibility, diagnosis, and monitoring efficacy of preventive or therapeutic strategies. Aptamers, comprising single-stranded or double-stranded DNA or RNA, can serve as biomarkers of disease or biological states. Aptamers can bind to specific epitopes on macromolecules by virtue of their three dimensional structures and, much like antibodies, aptamers can be used to target specific epitopes on the basis of their molecular shape. The Systematic Evolution of Ligands by EXponential enrichment (SELEX) is the approach used to select high affinity aptamers for specific macromolecular targets from among the >10^13^ oligomers comprising typical random oligomer libraries. In the present study, we used live cell-based SELEX to identify DNA aptamers which recognize cell surface differences between HPV-transformed cervical carcinoma cancer cells and isogenic, nontumorigenic, revertant cell lines.

**Methodology/Principal Findings:**

Whole-cell SELEX methodology was adapted for use with adherent cell lines (which we termed Adherent Cell-SELEX (AC-SELEX)). Using this approach, we identified high affinity aptamers (nanomolar range K_d_) to epitopes specific to the cell surface of two nontumorigenic, nontumorigenic revertants derived from the human cervical cancer HeLa cell line, and demonstrated the loss of these epitopes in another human papillomavirus transformed cervical cancer cell line (SiHa). We also performed preliminary investigation of the aptamer epitopes and their binding characteristics.

**Conclusions/Significance:**

Using AC-SELEX we have generated several aptamers that have high affinity and specificity to the nontumorigenic, revertant of HPV-transformed cervical cancer cells. These aptamers can be used to identify new biomarkers that are related to carcinogenesis. Panels of aptamers, such as these may be useful in predicting the tumorigenic potential and properties of cancer biopsies and aid in the effective management of pathological conditions (diagnosis, predicted outcome, and treatment options).

## Introduction

Cervical cancer is the second most common cancer affecting women worldwide [1]. More than 90% of cervical cancers are caused by HPV, and approximately 10,800 new cases of HPV-related cervical cancer are diagnosed in the United States (US) annually [2]. More than 100 HPV types have been classified, the most oncogenic of which are HPV16 and HPV18. HPV type 16 (HPV-16) is associated with more than 50% of cervical cancers worldwide [3]. A recent study showed that HPV infection is also associated with sexual activity and is the leading cause of the increasing incidence of oropharyngeal cancer in the US [4]. Thus, despite the availability of a preventive vaccine for HPV infection [5], there remains a significant need for the development of biomarkers that are specifically associated with HPV carcinogenesis rather than simply infection. These transformation specific biomarkers would be useful for studies of disease progression, prevention, and/or response to therapy. Here we describe the development of an aptamer-based approach to identifying biomarkers of HPV-mediated cell transformation.

The concept of aptamers arose through the observation that macromolecules with different primary molecular structures will each adopt a unique, three-dimensional configuration, each of which will have unique affinities for binding to, and forming complexes with other molecules [6]. It is in fact the same type of sequence-dependent structural variations that is the basis for specific binding of antibodies to antigen epitopes. Over the past ∼20 years, the concept of structure-based binding has been exploited to develop aptamer libraries, sets of macromolecules with randomized sequences of nucleic acids (RNA or DNA). Aptamer libraries are then screened for their ability to bind preferentially to specific molecules or macromolecules using SELEX (Systematic Evolution of Ligands by EXponential enrichment), which combines aptamer enrichment through iterative selection by affinity binding and polymerase chain reaction (PCR), to discover high affinity, feature-specific double-stranded or single-stranded RNA or DNA [6], [7]. While aptamers can have affinities comparable to those of monoclonal antibodies they have several advantages. Aptamers can be produced *in vitro* and hence do not require immunization, cell fusion, or fermentation to produce mass quantities. Once the nucleic acid sequence of a functional aptamer is determined, large quantities of the specific oligomer can be produced by chemical or enzymatic synthesis at low cost and high efficiency.

A more recent application is cell-SELEX, where aptamers that recognize specific molecules on the surface of the cells are evolved by repeated amplification and binding to living cells [8]. In this approach aptamers can be used to identify a wide variety of molecules including nucleic acids, proteins lipids, carbohydrates as well as posttranslational modification of such molecules that are on the surface of the exposed cells. Significantly, cell-SELEX offers the unique ability to discover unknown or unidentified biomarkers associated with a specific cell type, developmental stage, exposure, infection, biological state or disease. Cell-SELEX has been successfully utilized in non-adherent cell cultures to identify aptamers which can recognize leukemia cells in biological specimens [8], [9]. In the present study we adapted whole cell-SELEX for use on adherent cell lines (target and non-target), a process which we termed Adherent Cell-SELEX (AC-SELEX) for simplicity. Using AC-SELEX, we evolved aptamers that can distinguish human papillomavirus (HPV)-transformed HeLa cervical carcinoma cells from non-tumorigenic revertants of HeLa cells (HF) that retain a functional, integrated HPV genome [10], [11]. The HF revertant cell line was previously derived in our laboratory from HeLa cells exposed to a single mutagenic dose of, ethylmethylsulfonate (EMS), followed by selection of clones from cells that lost the prolonged Rhodamine 123 retention time characteristic of most cancer cells [12]. Relative to the parental HeLa cells, HF cells have a non-transformed phenotype, decreased cloning efficiency in soft agar and are non-tumorigenic in athymic nude mice [11]. HF×HeLa cell hybrid cells generated by cell fusion showed significant reduction in cloning efficiency in soft agar, suggesting the activation of a dominant tumor suppressor gene in the HF cells and molecular analysis indicated reactivation of the p53 tumor suppressor gene in HF cells, but without the loss, rearrangement, or reduction in the expression of the HPV E6/E7 oncoproteins [10], [11]. Given that HF cells are isogenic with HeLa cells, differences in their gene expression patterns are likely to be associated with loss of the tumorigenic phenotype, making this the ideal cell pair in which to search for biomarkers of HPV-mediated cell transformation.

Here we report on the discovery of single-stranded DNA aptamers with high affinity for epitopes that are enriched on the surface of the adherent, nontumorigenic HF cell line relative to the tumorigenic HeLa cell line. The ability of the aptamers to detect biomarkers lost during HPV-mediated cell transformation was validated in the SiHa cervical carcinoma cell line. The results of this study indicate that aptamers can be used to elucidate candidate biomarkers for cellular changes associated with and/or contributing to generation of a non-tumorigenic phenotype in HPV-infected cells.

## Results

### Adherent Cell-SELEX and Identification of Target-Cell Specific Aptamer Candidates

**Figure 1:**
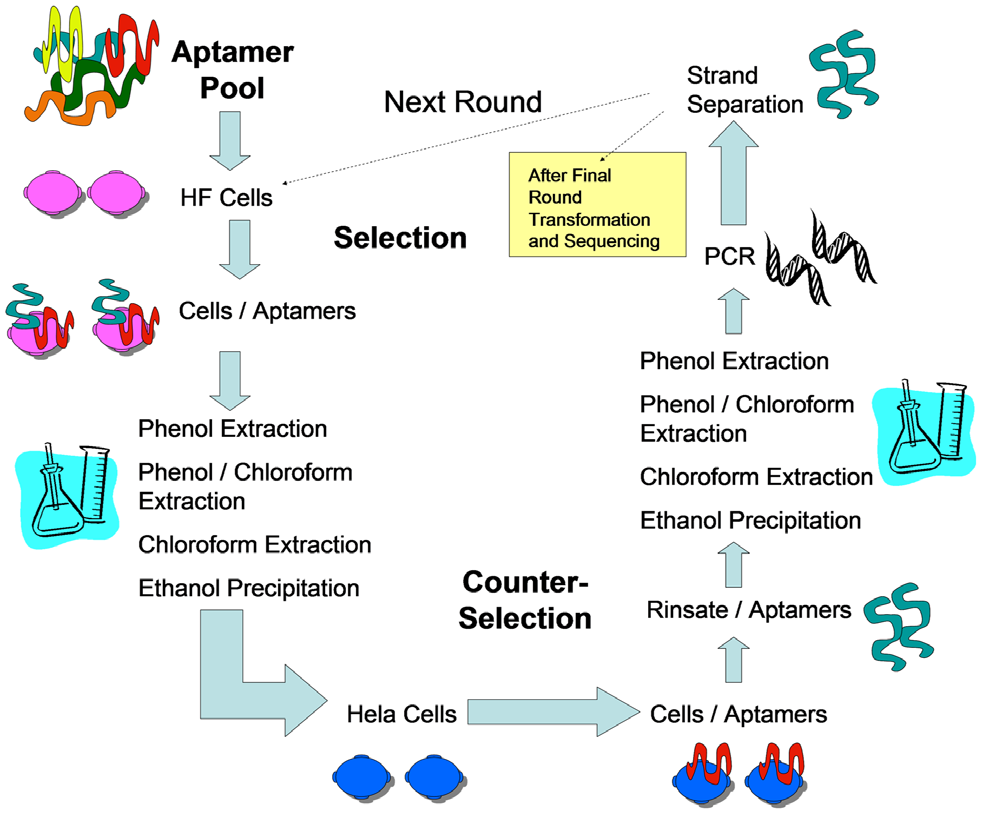
Schematic of Adherent Cell-SELEX (AC-SELEX) Procedure. A starting library of >10^15^ aptamers was utilized and eighteen rounds of AC-SELEX were carried out to purify and amplify the aptamers which recognized epitopes present on HF cells and not present on HeLa cells. The starting library was first incubated with the HeLa cell line and the unbound aptamers were recovered and utilized for the AC-SELEX procedure.

**Figure 2:**
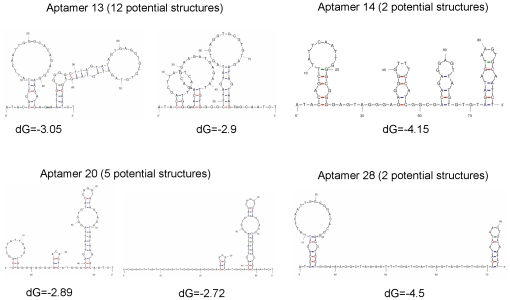
Predicted aptamer secondary structures. The secondary structures for each of the aptamers investigated. The structure(s) with the lowest free energy (dG) are presented. Secondary structure predictions were determined using UNAfold software and are sir_graph ® output [13].

To adapt the whole cell-SELEX to adherent cells in culture, we utlized the procedure outlined in Figure 1. Aptamers were targeted to the nontumorigenic HF cell line, using isogenic HeLa cells for negative counter-selection. After eighteen cycles of positive/negative selection we identified molecular probes that were highly specific for the revertant HF cell line (Table 1). We sequenced eleven aptamer candidates and of these, four were randomly chosen for further DNA sequence analysis and binding studies (**bold** in Table 1). Since aptamers can be thought of as comprising “shape" libraries, we generated potential secondary structures for each of the selected aptamers and calculated the relative free energies for each of the predicted structures [13] (Figure 2).

**Table 1:** Aptamer sequences.

Aptamer ID	Aptamer Sequences (random region)
10	5′- GGGCAGGACTAGGGTAACGAGGTCGCATTTGGAGCAGGTG CAGGTGCGGTGT - 3′
**13**	5′ - GGGCACAGACGGAAGATGAGAATTGTGGGGCTTAGTATAG TGAGGTGCGTGT - 3′
**14**	5′ - GGGCGGGGAGTAGGGAGAGGGGTTTCCATCGGCGACAG AGGAGTTATGTGTGT - 3′
17	5′ - GGGGAGGGCGGTGATTAGAAGATTGCCTCTGATGAGGTAG GTCGGTGTGTGT - 3′
**20**	5′ - GGGGAGGGAGACACAGTCATGGAGCAGTTATTAGGGTGT ACCGGGTGTAGT - 3′
21	5′ - GGGGGGGAGAACGTAGTATGCATGGCGGTAATGAATGCTT GTAGTGCGTGGT - 3′
24	5′ - GGGGACAGGTAACAGGTGGGCAGTGTTGATCGGGGTGTTC GGTTTGCTGTGG - 3′
26	5′ - GGGGCGGAGTGGCTAGCGGGCAGCAAAAGGATGAGTCCCT GGAGTTATTGCC - 3′
27	5′ - GGGGAGCGGCTAGGATTGGGTGTGTGTAGCTGGATGAGGT CAATGTCGTGCT - 3′
**28**	5′ - GGGGGACACGGAGGTGGTGGAAAGGCTAAGATTTGATGAT GAGTAGTGTGGT - 3′
29	5′ - GGGGACGCGGGTGTACTAAGACAATTCAGTGCGATGGTAG TGGTGTGGCGGT - 3′

All Aptamer sequences are flanked by the primer sequences: 5′ – ATA CCA GCT TAT TCA ATT – N_52_ - AGA TAG TAA GTG CAA TCT – 3′. Aptamers in **bold** were randomly chosen for further characterization.

### Aptamer Binding Sites on the Target Cells

**Figure 3:**
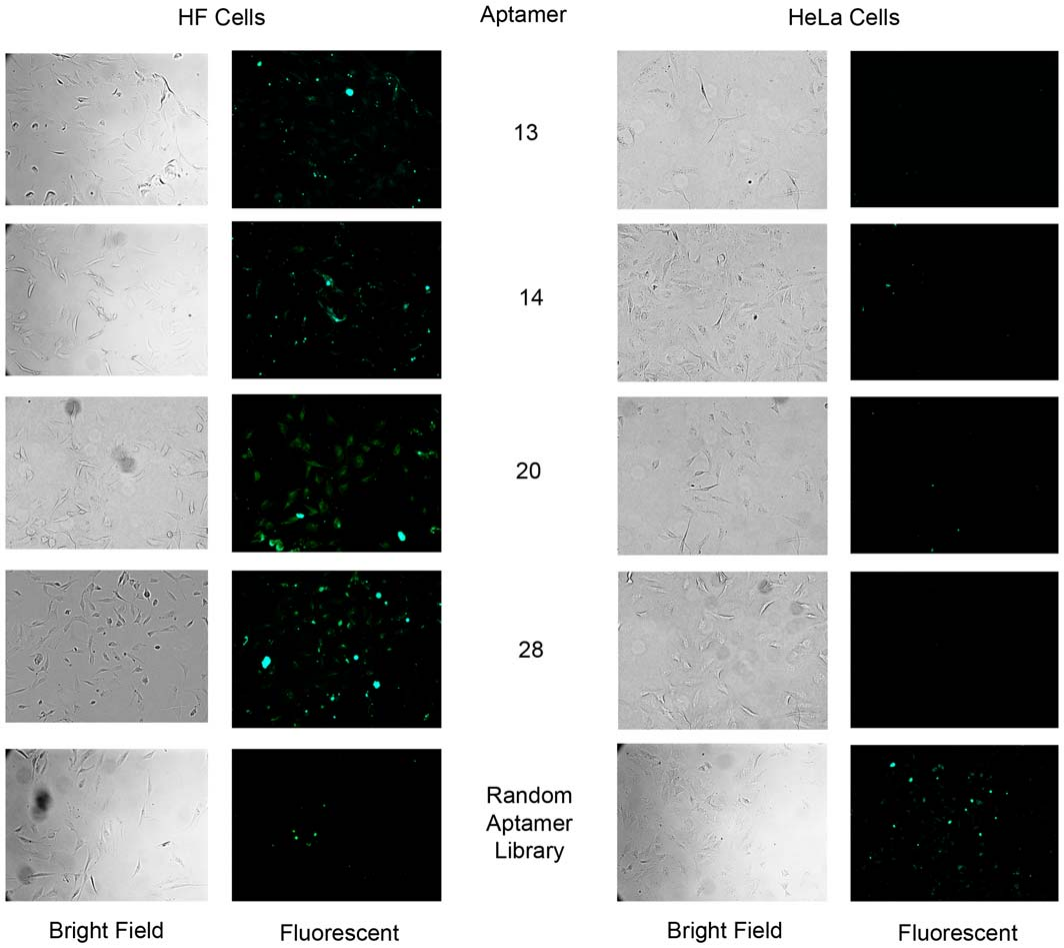
The target (HF) and non-target (HeLa) cell lines after incubation with FAM labeled aptamers. Images of HF and HeLa cells incubated with fluorescently tagged aptamers. The aptamers are specific for the non-tumorigenic HF cell line. They recognize epitopes on or in the HF cells and do not recognize their binding sites on the tumorigenic HeLa cells. All images are 40× and are of cells within 24 hours after incubation with 2000 nM aptamers and subsequent fixation and coverslipping with Aquamount.

Once we obtained the nucleotide sequences for our target-cell specific aptamers, the corresponding single-stranded DNA oligonucleotides were synthesized with fluorescent FAM tags. The labeled aptamers were then incubated with the HF cells to determine the cellular localization of their binding sites (Figure 3). The FAM conjugated aptamers were also incubated with the parental HeLa cells to verify differential binding.

**Figure 4:**
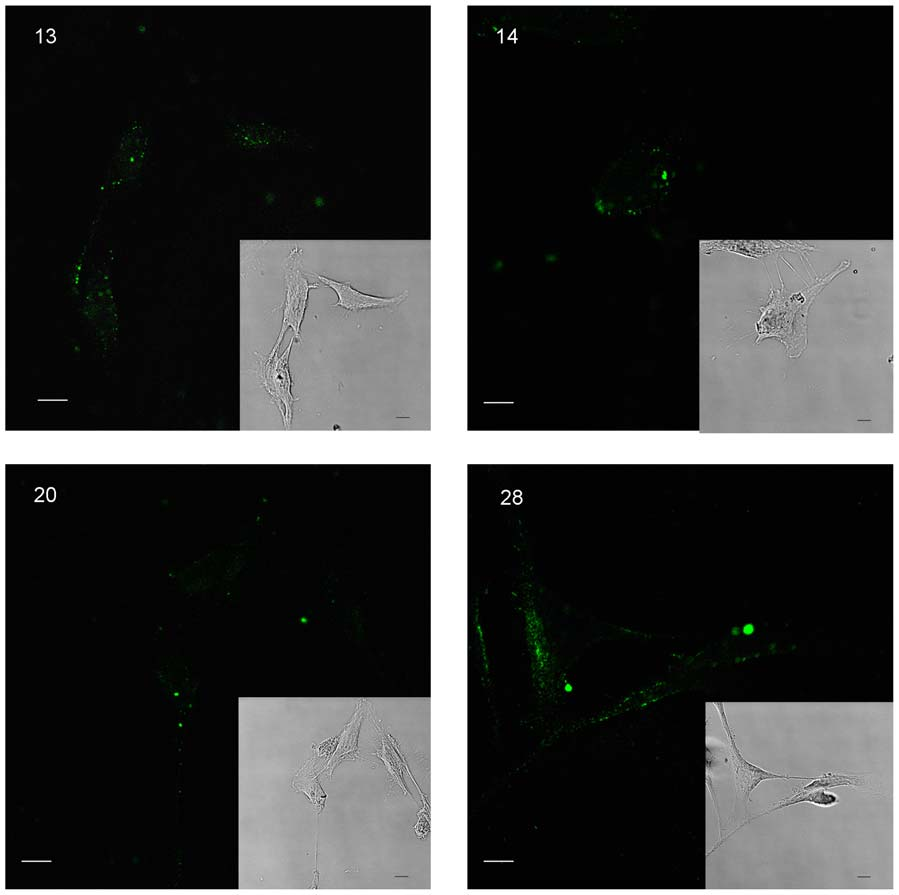
Confocal images of aptamer-HF cell binding sites. Images of HF cells with FAM labeled aptamers. Aptamers 13, 20 and 28 appear to be binding solely to the cell surfaces, while aptamer 14 is also being internalized into the cell cytoplasm. All images are 40× and are of cells within 24 hours after incubation with 2000 nM aptamers and subsequent fixation and coverslipping with Aquamount.

Confocal imaging and image reconstruction using z-stacks was used to determine the cellular localization of aptamer-epitope complexes (Figure 4). The binding sites of aptamers 13, 14, 20, and 28 appeared to be located on the cell surface. Aptamer 14, however, also appears to be binding to the perinuclear region within the cell, which is consistent with binding of aptamer 14 to epitopes on the cell surface followed by transport to the perinuclear region. Although the mechanism by which this oligomer entered the cell is unclear, pre-incubation of the HF cells with proteinases (proteinase K or trypsin for 2 or 10 minutes prior to aptamer binding) did not affect the ability of the cells to internalize the FAM labeled aptamers. By contrast, proteinase treatment significantly reduced the ability of aptamers 13, 20 and 28 to bind to the cells, suggesting that they were associated with epitopes on cell surface proteins.

### Aptamer Binding Characteristics

**Figure 5:**
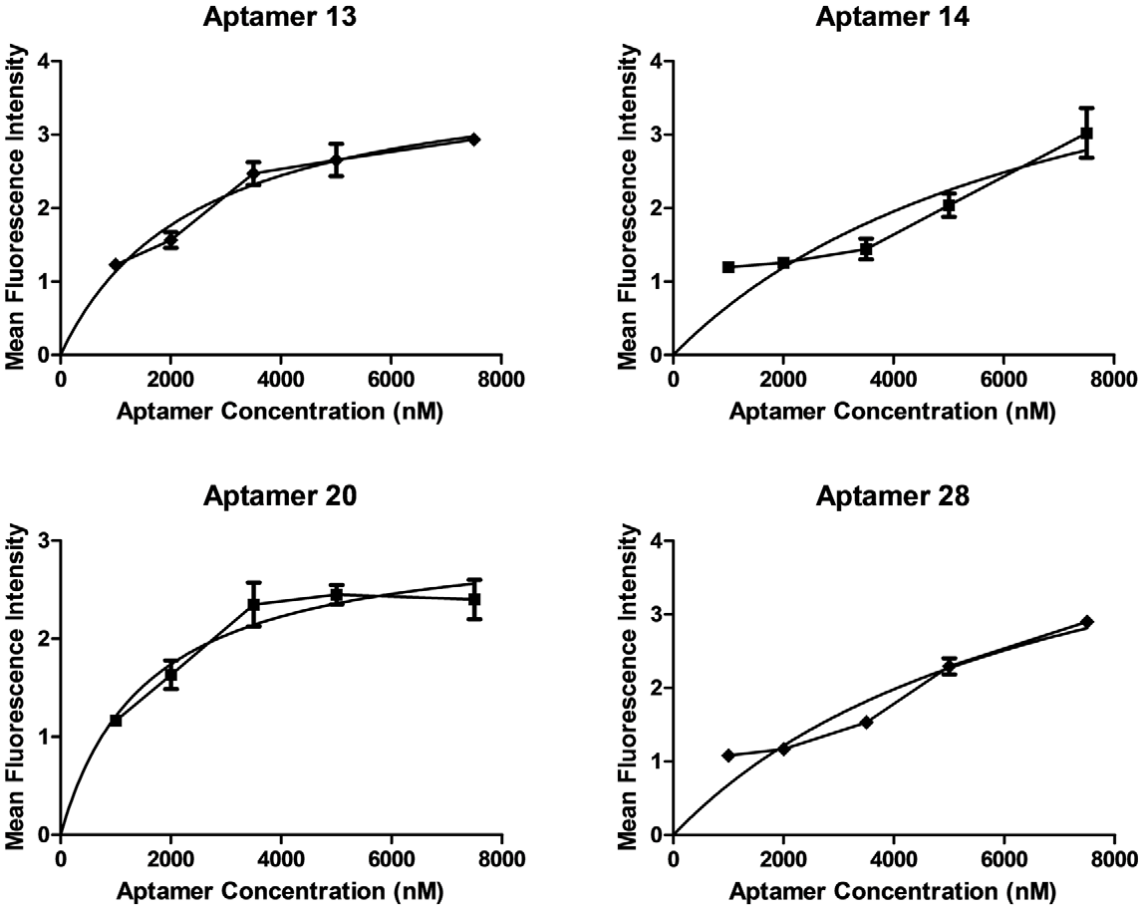
Binding equilibrium for aptamers and their HF cell epitopes. Fluorescence quantification of FAM-labeled aptamers bound to HF cells. Aptamer binding curves (line and data points) were generated by graphing the average fluorescence with the error bars representing the standard error of the mean for each data point (n = 4 per data point). These data were fitted to the model Y = Bmax*X(Kd+X) (solid line) which was generated using nonlinear regression analysis for one site specific binding: aptamer 13 (K_d_ = 2.5±0.5 nM); aptamer 14 (K_d_ = 7.1±0.4 nM); aptamer 20 (K_d_ = 1.6±0.4 nM); and aptamer 28 (K_d_ = 6.9±0.2 nM).

To determine the binding equilibrium of the aptamers to their HF epitopes, we incubated HF cells with incremental concentrations of the FAM tagged aptamers and measured the fluorescence of individual cells (Figure 5). Based on our analysis, all aptamers evaluated showed high affinity binding to their epitopes: Aptamer 13 (K_d_ = 2.5±0.5 nM); Aptamer 14 (K_d_ = 7.1±0.4 nM); aptamer 20 (K_d_ = 1.6±0.4 nM); and Aptamer 28 (K_d_ = 6.9±0.2 nM).

### Aptamer Specificity for the Non-tumorigenic, HPV-transformed Cervical Cancer Cell Line

**Figure 6:**
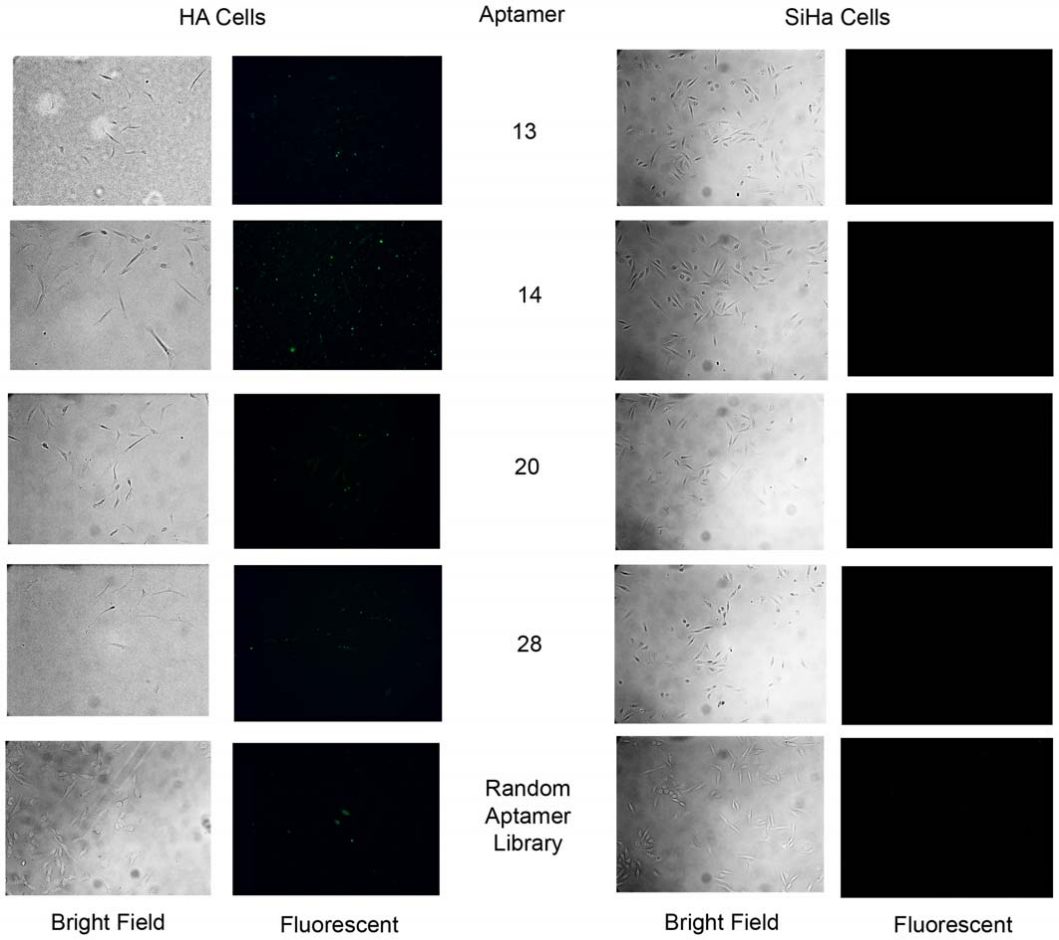
The nontumorigenic, HA and tumorigenic SiHa cervical cancer cells after aptamer incubation. Images depicting the non-transformed revertant, HA cell line and the HPV-transformed cervical cancer cell line, SiHa, after incubation with FAM labeled aptamers. The aptamers generated to target the non-transformed revertant, HF cell line also recognize the non-transformed revertant, HA cell line. Also notice that these aptamers do not recognize epitopes on the SiHa cervical cancer cell line, similar to the results we observed with the HeLa cervical cancer cell line. These results are consistent with the hypothesis that these aptamers are recognizing epitopes that are lost as a result of transformation of cervical epithelial cells by HPV. All images are 40× and are of cells within 24 hours after incubation with 2000 nM aptamers and subsequent fixation and coverslipping with Aquamount.

To investigate whether the aptamers evolved for binding to non-tumorigenic HF cells were recognizing cell-surface differences specific to non-tumorigenic revertants, we also examined binding to an independent clone of non-tumorigenic revertants of HeLa cells (HA) and to another HPV-transformed cervical carcinoma cell line (SiHa). Incubations of the FAM tagged aptamers with the HA and SiHa cell lines (Figure 6) illustrated that the aptamers specific for the non-transformed HF cells also bound to the HA revertant clone but did not bind to the transformed SiHa cells. These results indicated that the aptamers evolved against HF revertant cells are specific for epitopes that were lost during HPV-induced transformation of the cervical cells. Studies using the aptamer reagents for affinity chromatography are in progress to purify and identify the macromolecules harboring the epitopes.

## Discussion

Highly sensitive and specific biomarkers are extremely useful for diagnosis, treatment and prognosis of cancer cases. Cell-SELEX is an *in vitro* procedure through which aptamers can be chosen by their ability to bind differentially to cells [14], without any prior knowledge of cellular changes. Aptamers can bind with high affinity and specificity to a variety of molecules [15], [16], [17] and offer a unique alternative to antibodies which are much larger, easily degraded, costly and time-intensive to develop, and require the use of animals [18]. Nucleic acid based-aptamers are easily synthesized and can undergo reversible denaturation for PCR amplification. They can also recognize small molecules like toxins [19], [20] and molecules that are not non-immunogenic complexes [21] and aptamers can be easily modified with reporter molecules or other molecules to enhance their properties (i.e. half life) [18]. Aptamers also adopt unique sequence-dependent three-dimensional shapes, so they are in a sense, shape libraries and they may bind to a cell based on their sequence, conformation and/or charge through interactions with binding pockets, hydrogen bonds, stacking of aromatic rings, van der Waals forces, or a combination of these [22]. Aptamers can interfere with protein-protein interactions, compete for and interfere with binding sites, interfere with virus-cell attachment, transcription and mRNA translation [23]. Owing to their small size, aptamers may also be able to access epitopes that are normally blocked/hidden to other probes/therapeutics. These desirable properties, afford aptamers the ability to serve as therapeutic agents [21], [24], [25], [26], [27].

Aptamers are already being utilized in cancer biology and cancer medicine [28], [29], [30]. Using cell-SELEX, investigators have identified high affinity aptamers which increase the sensitivity of pancreatic cancer to chemotherapeutics [31], deliver therapeutics into cancer cells [32] or recognize cancer cells in biological specimens [8], [9]. SELEX has also enabled researchers to identify aptamers which target transformation-specific antigens, such as the HPV-16 E6 and E7 oncoproteins, in extracts of cervical cancer cells [33], [34]. Here we show that AC-SELEX can also be used to identify cell surface markers that are altered during HPV-induced cell transformation.

To evolve aptamers that differentiate HPV-transformed cells from non-transformed cervical cell without *a prior* selection of epitopes or targets, we adapted whole cell-SELEX for use on adherent cell lines (Figure 1). Using this approach, we identified and independently validated aptamers which have high affinity for proteins or cell surface antigens that are specific to and present on the surface of nontumorigenic revertants of HeLa cells, but are not present on HPV transformed cervical carcinoma cell lines (Figure 3). Importantly, two independently derived revertant cell lines (HF and HA) continue to express the HPV E6 and E7 oncoproteins, but unlike the parental HeLa cells, the HF and HA cell lines have a non-transformed morphology, contact inhibited growth, significantly reduced cloning efficiency in soft agar, and the inability to form tumors in nude mice [10], [11]. In a single screen, we were able to identify eleven unique aptamer sequences that specifically recognize and bind to ligands present on the nontransformed revertant HF cells, but not on the HeLa cells (Table 1). Based on our results, aptamers 13, 20 and 28 bind to HF specific cell surface epitopes, while aptamer 14 is binding to the cell surface and being internalized into the cell cytoplasm (Figure 4). The ability to abolish aptamer binding with pretreatment of cells with protease suggested that three of the four aptamers tested recognized epitopes on cell surface proteins. Aptamer 14 was able to enter the cells independent of cell surface protein binding, or was recognizing a protease resistant cell-surface epitope that internalized the aptamer complex. Together these findings indicated that AC-SELEX was able to evolve aptamers that recognize changes in cell surface macromolecules which distinguish the tumorigenic from non-tumorigenic HPV-infected cells, suggesting utility as diagnostic probes.

To explore their prognostic potential, aptamers 13, 14, 20 and 28 were tested with an independent HPV-positive cervical cancer cell line, SiHa, and an additional, non-tumorigenic revertant cell line, HA. Significantly, we observed that the aptamers did recognize their epitopes on non-tumorigenic HA cells, but did not bind to the tumorigenic SiHa cells (Figure 6), supporting the hypothesis that the aptamers recognize markers lost during HPV-mediated transformation of cervical epithelial cells. Since the HF, HA and HeLa cell lines are isogenic, we expect the differences in gene expression not related to transformation to be minimal, and therefore that the aptamers evolved are likely to recognize important biomarkers and epitopes indicative of cervical cell tumorigenicity. These results indicate that aptamers generated by AC-SELEX have the ability to preferentially recognize highly tumorigenic cervical cancer cell lines relative to non-tumorigenic cervical cancer cell line revertants.

In summary, the present study demonstrated the feasibility of Adherent Cell-SELEX as well as its utility in detecting epitopes that are lost as a result of transformation of cervical epithelial cells by HPV. Panels of aptamers such as these, evolved against different tumor cell lines to distinguish cancer types and subtypes may be useful used to relate diagnosis and prognosis and to determine chemotherapy and disease susceptibility (i.e. genetic differences).

## Materials and Methods

### Cell Culture

The early passage ATCC CCL2 clone of Hela cells as well as the SiHa cervical carcinoma cell line were obtained from the American Type Culture Collection. HF and HA cells were obtained from experiments performed previously in which selection of nontransformed revertants was obtained based on the loss of rhodamine 123 from the cell mitochondria [11]. HeLa, HF, HA and SiHa cells were cultured in Dulbecco's Modified Eagle Medium (Gibco) with 10% fetal bovine serum and 1% penicillin-streptomycin solution.

### SELEX Library and Primers

Single-stranded DNA sequences with 52 random nucleotides flanked by known 18-nt primer sites were obtained from Integrated DNA Technologies. The primer sequences were 5′-ATACCAGCTTATTCAATT and 5′-AGA TAG TAA GTG CAA TCT. The FAM tagged primers were created as 5′-FAM- ATACCAGCTTATTCAATT and the biotin tagged primers were created as 5′-Biotin-AGATTGCACTTACTATCT.

### SELEX Procedures

Before beginning, a starting library of approximately 1.6×10^15^ aptamers was incubated with the non-target HeLa cells and then the unbound aptamers were recovered and prepared for the first round of AC-SELEX. Prior to each round, aptamers were denatured by heating at 95°C for 5 minutes and then briefly cooled on ice for 2 minutes. For each round of incubations, 3×10^5^ HF cells were rinsed with PBS and incubated on ice with the aptamer library in binding buffer (1% BSA in PBS with salts) under gentle agitation. The cells were then rinsed with PBS and the aptamers which bound to the HF cells were eluted through the addition of the elution buffer (100 mM NaCl, 10 mM Tris 7.5, 1 mM EDTA and 1% SDS) and recovered using the Hirt DNA prep method [35] which involved phenol extraction, phenol-chloroform extraction, chloroform extraction. We also incorporated a back-extraction step to ensure adequate aptamer recovery. The genomic DNA was then removed from the ethanol solution. Aptamers were recovered from solution by ethanol precipitation (3 M NaOAc in 100% ethanol) and allowed to settle overnight at −20°C. The resulting sample was then centrifuged and the pellet rinsed twice with 70% ethanol and allowed to dry. The pellet was then reconstituted in binding buffer and incubated with 10^6^ HeLa cells for aptamer counter-selection. The cells were then rinsed with PBS and the nonbinding aptamers were recovered from this rinsate again utilizing the Hirt method, including a back-extraction step, followed by ethanol precipitation of aptamers. The resulting aptamer libraries were then amplified via PCR with a biotin-tagged primer and a non-biotin-tagged primer (Integrated DNA Technologies), and the resulting double-stranded aptamers were then separated using streptavidin coated beads and column centrifugation to separate the unwanted biotin-tagged sequence from the desired aptamer sequence (See **PCR and Aptamer Strand Separation**). The resulting single-stranded aptamers were then precipitated and used for the next round of AC-SELEX.

### PCR and Aptamer Strand Separation

Aptamers resulting from the counter-selection step were reconstituted with elution buffer (100 mM NaCl, 10 mM Tris 7.5, 1 mM EDTA and 1% SDS) and amplified via PCR with biotin-tagged primers (94°C for 30 sec, 46°C for 30 sec, 72°C for 30 sec) on an Eppendorf-AG 22331 thermocycler (Hamburg, Germany). The conditions for PCR amplification of aptamer libraries differs from that of homogeneous DNA. PCR was optimized around performing 18 rounds of AC-SELEX. Research with a random nucleotide of this similar length has shown that product formation reaches a maximum at 18 rounds and if more than 20 rounds of PCR are performed, by-products begin to form and there is a risk of complete loss of aptamer product [36]. The resulting biotin-tagged double-stranded aptamers were then boiled for 5 minutes, flash cooled in ice water for 3 minutes, and incubated with streptavidin coated beads in column binding buffer (20 mM NaPO4, 150 mM NaCl) prior to column centrifugation. The resulting single-stranded aptamers were precipitated from the column flow through via ethanol precipitation. The aptamer pellet was then reconstituted in PBS with 1% BSA and this aptamer solution was used for the next round of AC-SELEX.

### Aptamer Cloning

After 18 rounds of AC-SELEX, the resulting aptamer pool was PCR-amplified with unmodified primers. The resulting aptamer library was then ligated into plasmids and cloned into Escherichia coli using the *TOPO* TA *cloning kit* containing pCR®2.1-*TOPO*® (*Invitrogen*, K4500-01). The plasmids were purified using QIAprep Spin Miniprep (QIAGEN, 27106). The concentrations of the resulting purified plasmids were determined using the Nanodrop TM 1000 Spectrophotometer (Thermo Scientific). PCR followed by gel electrophoresis was then performed on the purified plasmid samples to ensure an insert of the proper length.

### Aptamer Sequencing

Double stranded aptamers were sequenced using the M13 forward and reverse primers provided in the TOPO 10 Cloning kit. Plasmids were sequenced by the University of Medicine and Dentistry of New Jersey DNA Core Facility (Piscataway, NJ).

### DNA Folding Predictions

The secondary structures of the single-stranded DNA aptamers were predicted using UNAfold mfold software (http://mfold.rna.albany.edu). The structures presented are output from sir_graph ® developed by D. Steward and M. Zuker [13].

### Imaging of Cell-Aptamer Complexes

FAM 5′-tagged aptamers were synthesized by Integrated DNA Technologies. Cells were cultured in chamber slides, grown overnight, rinsed with PBS and then allowed to incubate with the fluorescently labeled aptamer(s) in PBS with 1% BSA on ice for 45 minutes. Cells were then rinsed with PBS prior to fixation with 4% paraformaldehyde. The fixed cells were then rinsed with PBS and quickly with DNase free water and mounted using Aquamount (Polysciences, Inc.). Cell-bound aptamers were then imaged using a Leica confocal microscope.

### Fluorescence Quantification Analysis

The fluorescence of the aptamer-cell complexes were measured using Leica Lite software. Cells were imaged using a Leica confocal microscope and individually quantified using Leica Lite software. Four cells were quantified per data point after background subtraction. The average fluorescence is graphed with the error bars representing the standard error of the mean for each data point. Apparent Kd values for each aptamer were determined by nonlinear regression for on-site binding according to the equation: Y = Bmax*X/(Kd+X) using GraphPad Prism version 5.04 for Windows (GraphPad Software, La Jolla California USA, www.graphpad.com [37].

### Proteinase Treatment of Cells

We designed our AC-SELEX procedure to allow for aptamer binding to surface protein epitopes which may be sensitive to proteinases. In order to determine which aptamer epitopes were sensitive to proteases, we treated the target cells with proteases a minimum of 4 times for each proteinase and aptamer and determined aptamer binding. Cells were treated with 0.05% trypsin and 0.53 mM EDTA in HBSS or 0.1 mg/ml proteinase K in PBS at 37°C for 2 and 10 min. The proteinase treated target cells were then used for fluorescently labeled aptamer binding as described above.
